# A successful backward step correlates with hip flexion moment of supporting limb in elderly people

**DOI:** 10.1371/journal.pone.0190797

**Published:** 2018-01-05

**Authors:** Yahiko Takeuchi

**Affiliations:** Department of Rehabilitation Science, Faculty of Health Care Science, Chiba Prefectural University of Health Sciences, Chuoku, Chiba, Japan; Universitat de Valencia, SPAIN

## Abstract

**Purpose:**

The objective of this study was to determine the positional relationship between the center of mass (COM) and the center of pressure (COP) at the time of step landing, and to examine their relationship with the joint moments exerted by the supporting limb, with regard to factors of the successful backward step response.

**Methods:**

The study population comprised 8 community-dwelling elderly people that were observed to take successive multi steps after the landing of a backward stepping. Using a motion capture system and force plate, we measured the COM, COP and COM-COP deviation distance on landing during backward stepping. In addition, we measured the moment of the supporting limb joint during backward stepping. The multi-step data were compared with data from instances when only one step was taken (single-step). Variables that differed significantly between the single- and multi-step data were used as objective variables and the joint moments of the supporting limb were used as explanatory variables in single regression analyses.

**Results:**

The COM-COP deviation in the anteroposterior was significantly larger in the single-step. A regression analysis with COM-COP deviation as the objective variable obtained a significant regression equation in the hip flexion moment (R^2^ = 0.74).

**Conclusions:**

The hip flexion moment of supporting limb was shown to be a significant explanatory variable in both the PS and SS phases for the relationship with COM-COP distance. This study found that to create an appropriate backward step response after an external disturbance (i.e. the ability to stop after 1 step), posterior braking of the COM by a hip flexion moment are important during the single-limbed standing phase.

## Introduction

Falls can cause elderly people to require nursing care, and therefore, prevention of falls is an important issue in this population. Moreover, methods of reducing, as much as possible, the impact of falls are necessary in order to decrease the severity of fractures and other injuries [1,2]. The step response involves taking a step to support the body when balance is lost in the standing position. The step response is considered an important postural response for preventing falls and reducing the impact of falls in elderly people. The step response dramatically increases the base of support, providing more dynamic stability than postural responses that correct the body’s alignment along the axis of the ankle or the hip [3]. In addition, the degenerative kyphosis that occurs with age increases the likelihood of elderly people losing their balance backwards [4,5]; thus, it is necessary to focus on the backward step response.

Maki et al. reported that elderly people who took multiple successive steps after landing the step response were at high risk of falling [6]. When undertaking a kinematic investigation of the differences between stopping at one step and taking multiple successive steps after landing the backward step––that is, the factors that make up a successful step response––it is important to examine the relationship between the center of mass (COM) and the center of pressure (COP) of the stepping limb during step landing. In addition, attention needs to be paid to the supporting functions of the joints of the supporting limb to determine the factors needed to place the stepping leg in a more posterior position.

The objective of this study was to determine the positional relationship between the COM and the COP at the time of step landing, and to examine their relationship with the joint moments exerted by the supporting leg, with regard to factors of the successful backward step response.

## Methods

### Subjects

The study population comprised 8 subjects were selected from a group of 19 community-dwelling elderly people; subjects were observed to take successive steps after the landing of a backward step in response to an external disturbance in the posterior direction (multistep group). Subjects comprised 2 men and 6 women, with a mean age of 67.0 ± 3.1 years, a mean height of 159.8 ± 8.6 cm, and a mean weight of 59.7 ± 10.6 kg. Exclusion criteria assumed the orthopedic conditions associated with pain of the lower limbs and the neurological disorders that impair to backward step response.

Data relating to the multistep group was compared with data from instances when only one step was taken (single-step) in response to the external disturbance. The external disturbance was repeated several times. In 8 subjects, 7 subjects were able to stopping at single-step in the second trial, and 1 subject was able to stopping at single-step in the third trial.

All subjects received oral and written explanations of the purpose of the experiment, and gave their written consent. The study was approved by the ethics committee of Chiba Prefectural University of Health Sciences.

### Method of inducing a backward step response

Four force plates (AMTI, BP400600) were arranged such that there were 2 in front and 2 in back. The subjects stood with their left and right feet on the 2 anterior force plates. A disturbance in the posterior direction was applied, with reference to Push and Release Test [7,8]. A subject began by standing on the force plates, then moved their COM slowly backward until rested against the hands of an experiment administrator standing behind. The experiment administrator then checked the subject’s body alignment on the sagittal plane in a posture-correction mirror, set up to the side. When the line from the acromion and the greater trochanter became posterior to the heel, i.e. the COM line deviated backward from the base of support, the experiment administrator rapidly released their hands to induce a backward step response.

### Measurement devices

The COM during the backward step response was measured using a 3-dimensional (3D) motion analyzer (Motion Analysis, MAC3D) comprising 8 infrared cameras. Based on the Helen Hayes marker set, 19-mm diameter infrared reflective markers were placed on 25 points on the subjects’ bodies. The force plates were used to measure the COP. The point when the disturbance was applied was determined using a small accelerometer (Microstone, MA3-20AD) affixed to the dorsum of the experiment administrator’s hand. The data-sampling frequencies were 200 Hz for the infrared cameras and 1,000 Hz for the force plates and accelerometer. Signals from the force plates and accelerometer were uploaded via an Analog/Digital converter to a computing board on a personal computer and synced with the camera signals.

### Data analysis

#### (1) Definition of data-analysis segments (Fig 1)

**Fig 1:**
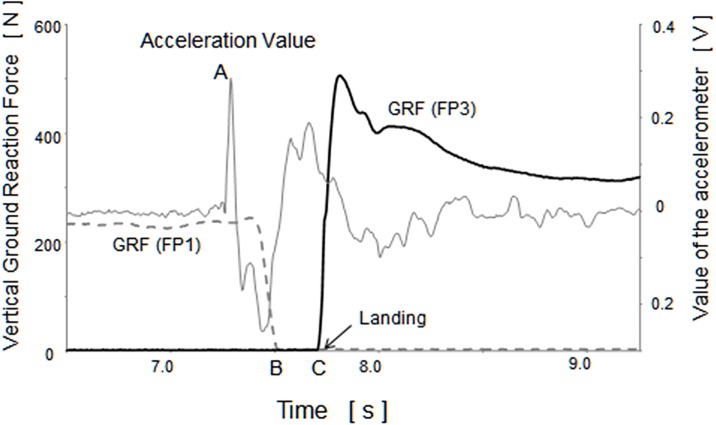
Definition of data-analysis segments from the ground reaction force and the acceleration value. A: initiation of the perturbation B: onset of the stepping C: Landing A-B: perturbation-stepping(PS) phase B-C: single-stance(SS) phase.

During the backward step response, from the time the disturbance was applied to when the stepping limb left the floor, was defined as the perturbation-stepping (PS) phase. The period from the time the stepping limb left the floor to immediately before its landing was defined as the single-stance (SS) phase.

#### (2) Step distance and COM-COP deviation distance

The COP immediately before takeoff of the stepping foot and the COP at landing were determined, and the distances between them were calculated anteroposterior and mediolateral. The COM position for the whole body was also calculated using the marker data obtained from the infrared cameras in 3D motion analysis software (C-motion, Visual3D). The position of the COM and of the COP of the stepping limb at the time of landing of the backward step was analyzed anteroposterior and mediolateral.

#### (3) Joint moments of the supporting limb

Using the motion analysis software, joint moments of the supporting limb during the PS and SS phases were calculated from the marker data and floor reaction force data. The joint moments were normalized by dividing them by each subject’s body weight.

### Statistical processing

Step distance was calculated from the change in position of the stepping limb’s COP and the distance of COM-COP deviation at the time of landing. Results of the single- and multi-step data were compared using a paired t-test.

Variables that differed significantly between the single- and multi-step data were used as objective variables and the joint moments of the supporting limb were used as explanatory variables in single regression analyses. The level of significance was set at 5%.

## Results

### Step distance and distance of COM and COP deviation

Tables 1 and 2 shows the step distances and the distances of deviation between the COM and the COP of the stepping limb in each direction in the single- and multi-step instances.

**Table 1:** Step distance and distance of COM-COP deviation of each subject in backward stepping.

Subject	Step distance [m]				COM-COP distance [m]			
	Single		Multi		Single		Multi	
	A-P	M-L	A-P	M-L	A-P	M-L	A-P	M-L
**A**	-0.22	0.02	-0.13	0.03	-0.07	0.12	0.03	0.11
**B**	-0.46	0.01	-0.33	0.03	-0.13	0.16	-0.02	0.14
**C**	-0.62	0.10	-0.66	0.10	-0.20	0.18	-0.07	0.16
**D**	-0.51	0.05	-0.30	0.02	-0.13	0.15	0.04	0.11
**E**	-0.26	0.004	-0.21	0.01	-0.10	0.12	-0.09	0.14
**F**	-0.37	0.03	-0.33	0.06	-0.06	0.13	-0.03	0.17
**G**	-0.46	0.14	-0.45	0.07	-0.12	0.19	-0.15	0.13
**H**	-0.42	0.02	-0.35	0.07	-0.23	0.15	-0.20	0.19

COM: Center of mass; COP: Center of pressure (stepping limb’s); A-P: Anterior-posterior; M-L: Medio-lateral

Backward distance: The distance that the COP is located backward to the COM

**Table 2:** Average value of Step distance and distance of COM-COP deviation.

Step	Step distance [m]		COM-COP distance [m]	
	A-P	M-L	A-P	M-L
Single	-0.41±0.13*	0.05±0.05	-0.13±0.06*	0.15±0.03
Multi	-0.35±0.16	0.05±0.03	-0.06±0.09	0.14±0.03

-: backward distance

*: p< 0.05

Values presented as mean ±SD.

COM: Center of mass; COP: Center of pressure (stepping limb’s); A-P: Anterior-posterior; M-L: Medio-lateral

Backward distance: The distance that the COP is located backward to the COM

The mean anteroposterior step distance was −0.41 ± 0.13 m in the single-step instances and −0.35 ± 0.16 m in the multi-step instances. The backward step distance was significantly larger in the single-step instances (p<0.05).

The mean anteroposterior distance of COM-COP deviation was −0.13 ± 0.06 m in the single-step instances and −0.06 ± 0.09 m in the multi-step instances. The deviation was significantly larger in the single-step instances (p<0.05).

Significant differences between the single- and multi-step instances were not observed regarding left-to-right step distance or left-to-right COM-COP deviation.

### Joint moments of the supporting limb

Table 3 shows the joint moments of the supporting limb during the PS and SS phases. Significant differences between the single- and multi-step instances were not observed for any joint moment.

**Table 3:** Joint moments of the supporting limb during the PS and SS phase.

・Perturbation-stepping (PS) phase									
Step	Hip joint [Nm/kg]			Knee joint [Nm/kg]			Ankle joint [Nm/kg]		
	Sagittal	Frontal	Horizontal	Sagittal	Frontal	Horizontal	Sagittal	Frontal	Horizontal
Single	0.69±0.25	0.21±0.67	-0.04±0.15	0.62±0.21	0.35±0.23	0.03±0.07	0.06±0.17	0.11±0.08	0.03±0.06
Multi	0.73±0.21	0.22±0.62	-0.10±0.22	0.63±0.19	0.35±0.26	0.03±0.07	0.10±0.18	0.13±0.09	0.03±0.05
・Single-stance (SS) phase									
Step	Hip joint [Nm/kg]			Knee joint [Nm/kg]			Ankle joint [Nm/kg]		
	Sagittal	Frontal	Horizontal	Sagittal	Frontal	Horizontal	Sagittal	Frontal	Horizontal
Single	0.76±0.28	0.25±0.86	-0.09±0.19	0.65±0.25	0.28±0.49	0.02±0.08	0.10±0.18	0.10±0.12	0.01±0.07
Multi	0.72±0.19	0.24±0.93	-0.10±0.24	0.57±0.24	0.29±0.49	-0.02±0.10	0.04±0.29	0.13±0.13	-0.003±0.07

Values presented as mean ±SD.

Hip joint sagittal: flexion +, frontal: abduction +, horizontal: external rotation +

Knee joint sagittal: extension +, frontal: valgus +, horizontal: external rotation +

Ankle joint sagittal: plantar flexion +, frontal: eversion +, horizontal: abduction +

### Relationships between step distance, COM-COP deviation, and joint moments

**Fig 2:**
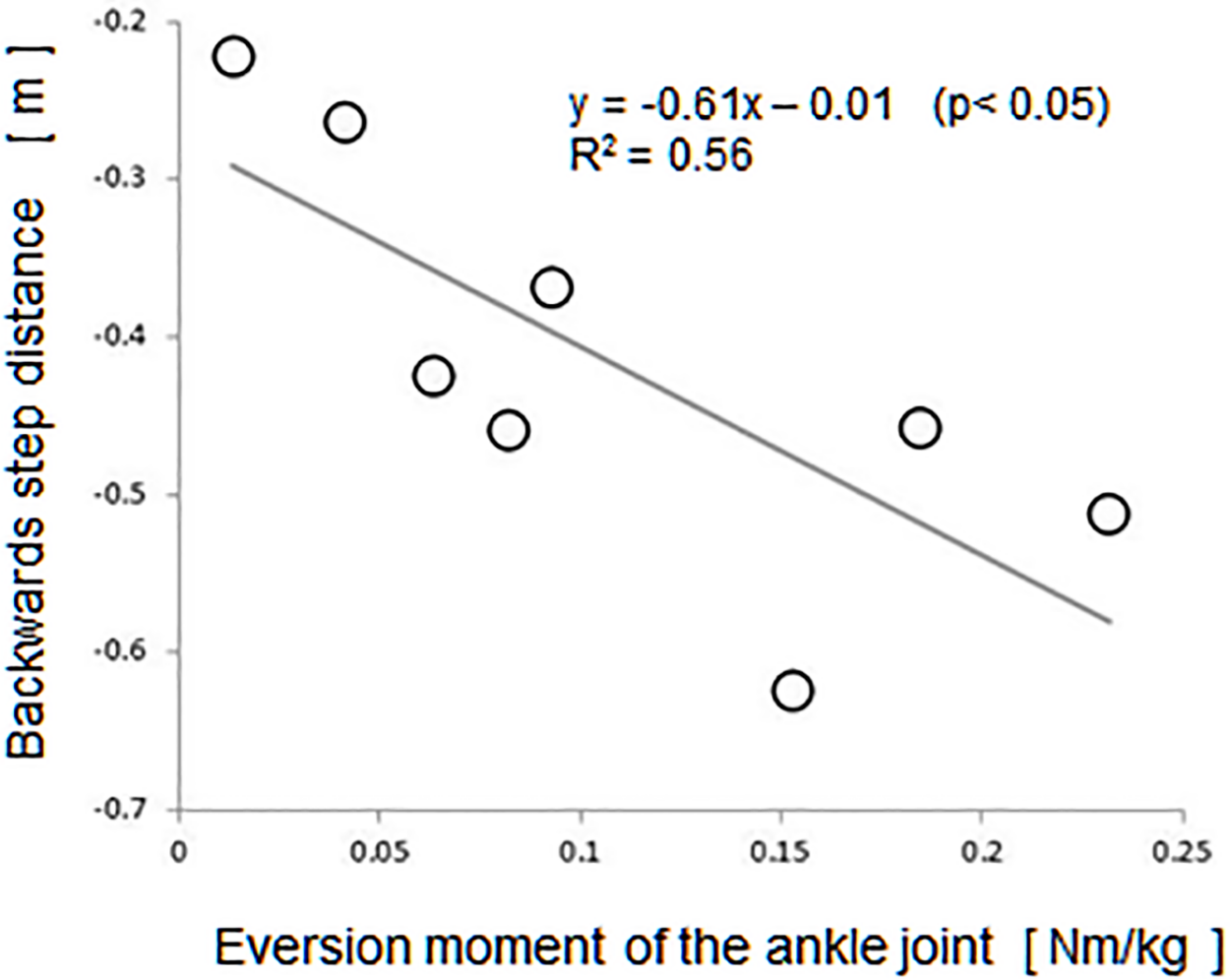
The relationship between backward step distance and the ankle eversion moment in the PS phase (single step).

A regression analysis with the step distance of the single-step instances as the objective variable obtained a significant regression equation in the PS phase with the knee valgus moment and the ankle eversion moment (y = -0.21x + 0.01, R^2^ = 0.74 and y = -0.16x -0.01, R^2^ = 0.56, respectively). Fig 2 shows the relationship between anteroposterior step distance and the ankle eversion moment in the PS phase. In the SS phase, a significant regression equation was obtained with the hip flexion/abduction moment (y = -0.34x -0.16, R^2^ = 0.52 / y = -0.12x -0.39, R^2^ = 0.61), the knee valgus moment (y = -0.21x -0.36, R^2^ = 0.62), and the ankle eversion moment (y = -0.96x -0.32, R^2^ = 0.75).

**Fig 3:**
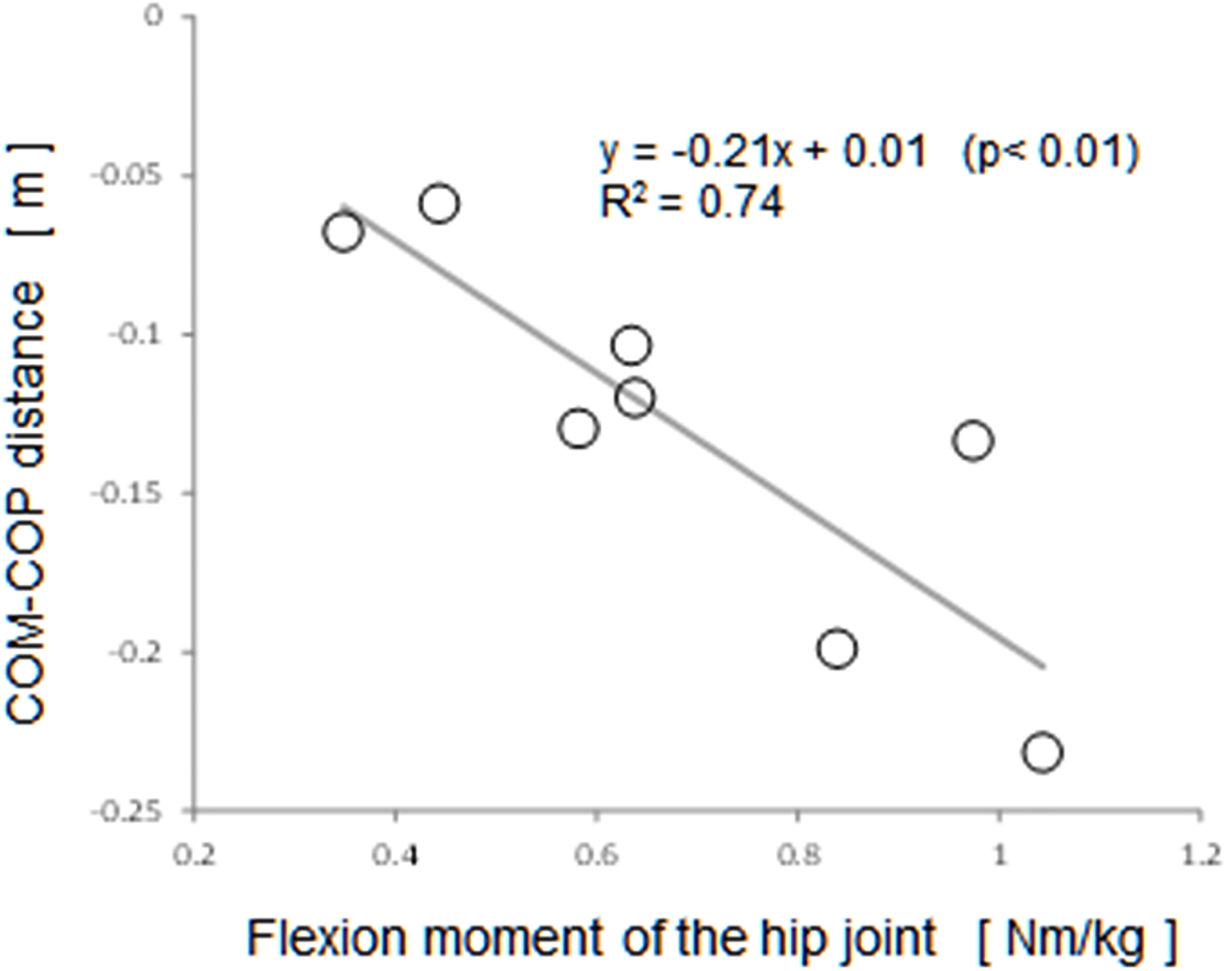
The relationship between COM-COP distance and the hip flexion moment in the PS phase (single step).

A regression analysis with COM-COP deviation as the objective variable obtained a significant regression equation in both the PS and SS phases with the hip flexion moment (y = -0.21x + 0.01, R^2^ = 0.74 and y = -0.16x -0.01, R^2^ = 0.56, respectively). Fig 3 shows the relationship between COM-COP deviation and the hip flexion moment in the PS phase.

## Discussion

Our examination of the single- and multi-step instances, centered around the distance traveled by the stepping limb, found significant differences in the backward stepping distance and in the deviation between the COM and the COP of the stepping foot at the time of landing. Moreover, these significant differences were only observed in the anteroposterior direction, not in the mediolateral direction. The results indicate that the ability to take a large step backward, and to position the COP of the stepping foot as posteriorly as possible with respect to the COM, are important factors in the successful backward step response.

Previous research [6, 9] on the step response in the anteroposterior direction found that frequent lateral instability occurs after landing in a substantial proportion of elderly subjects. In this study, some elderly people who used multiple steps after landing the backward step were observed to employ successive lateral steps to recover their balance. Because of this, it was hypothesized that we would observe differences between the single- and multi-step instances in the mediolateral distance and COM-COP distance in the present study; however, no such differences were found. The finding of significant differences only in the anteroposterior direction indicates that these subjects stabilized the COM by placing the COP, which is the point where floor reaction force was exerted, in a more posterior position, against the posterior/inferior acceleration that occurs in COM after application of the external disturbance.

Regression analyses of the backward step distance, COM-COP distance, and joint moments of the supporting limb, revealed relationships between them when the subjects were able to stop the step response at the single step. Significant explanatory variables for step distance included moments that contribute to lateral stability in both the PS and SS phases, such as the knee valgus moment and the ankle eversion moment. The hip flexion moment, which contributes to anterior stability, was a significant explanatory variable for COM-COP distance in both the PS and SS phases.

In the PS phase, there was a sudden shift in the COM to the supporting limb after the external disturbance, in preparation for takeoff of the stepping limb [10]. Exertion of the knee valgus and ankle eversion moments responded to this lateral movement of the COM, providing stability to the supporting limb and thereby allowing the stepping limb to move backward. Moreover, during the SS phase, the subject is standing only on the supporting limb, so the hip flexion/abduction moment was exerted to create more lateral and posterior stability, thereby creating step distance in the posterior direction.

The hip flexion moment of supporting limb was shown to be a significant explanatory variable in both the PS and SS phases for the relationship with COM-COP distance. The coefficient of determination (R^2^) was particularly large at 0.74 in the PS phase, when the external disturbance was applied posteriorly, indicating high predictive accuracy. After the external disturbance is applied in the PS phase, the COM accelerates backward and downward throughout both the PS and SS phase [11]. The regression analysis of the COM-COP distance and the hip flexion moment indicates that the flexion moment in the hip joint serves as a brake to posterior and inferior motion of the COM, from the pelvis above the thigh to the head, and in the joints of the upper limbs.

## Conclusions

In this study, we focused on the period of single-limbed standing from immediately after application of an external disturbance, until immediately before landing, during the backward step response. We analyzed relationships between factors that made up the successful step and joint moments of the supporting limb. The results suggest that to create an appropriate backward step response after an external disturbance (i.e. the ability to stop after 1 step), posterior braking of the COM by a hip flexion moment, and expanding the step distance via moments of lower limb joints on the frontal plane, are important during the single-limbed standing phase.

## Supporting information

S1 File Step distance and distance of COM-COP deviation of each subjects. (CSV)

S2 File Joint moments of the supporting limb of each subjects during the perturbation-stepping (PS) and single-stance (SS) phase. (CSV)

## References

[1] Cumming RG, Klineberg RJ. Fall frequency and characteristics and the risk of hip fractures. J Am Geriatr Soc. 1994; 42: 774–778. 8014355 10.1111/j.1532-5415.1994.tb06540.x

[2] Rogers MW, Johnson ME, Martinez KM, Mille ML, Hedman LD. Step training improves the speed of voluntary step initiation in aging. J Gerontol A Biol Sci Med Sci. 2003; 58: 46–51. 12560410 10.1093/gerona/58.1.m46

[3] Maki BE, McIlroy WE. Control of compensatory stepping reactions: age-related impairment and the potential for remedial intervention. Physiotherapy Theory Pract. 1999; 15: 69–90.

[4] Sinaki M, Brey RH, Huqhes CA, Larson DR, Kaufman KR. Balance disorder and increased risk of falls in osteoporosis and kyphosis: significance of kyphotic posture and muscle strength. Osteoporos Int. 2005; 16: 1004–1010. doi: 10.1007/s00198-004-1791-2 15549266 10.1007/s00198-004-1791-2

[5] Choi CJ, Lim HW, Park MK, Cho JG, Im GJ, Chae SW. Does the kyphotic change decrease the risk of fall?. Clin Exp Otorhinolaryngol. 2011; 4: 118–121. doi: 10.3342/ceo.2011.4.3.118 21949576 10.3342/ceo.2011.4.3.118 PMC3173701

[6] Maki BE, McIlroy WE. Control of rapid limb movements for balance recovery: age related changes and implications for fall prevention. Age and Aging. 2006; 9 35 Suppl 2: ii12–7. 10.1093/ageing/afl078 16926197

[7] Jacobs JV, Horak FB, Van Tran K, Nutt JG. An alternative clinical postural stability test for patients with Parkinson’s disease. J Neurol. 2006; 253: 1404–1413. doi: 10.1007/s00415-006-0224-x 16788773 10.1007/s00415-006-0224-x

[8] Horak FB, Wrisley DM, Frank J. The Balance Evaluation Systems Test (BESTest) to differentiate balance deficits. Physical Therapy. 2009; 89: 484–498. doi: 10.2522/ptj.20080071 19329772 10.2522/ptj.20080071 PMC2676433

[9] McIlroy WE, Maki BE. Age-related change in compensatory stepping in response to unpredictable perturbations. J Gerontol. 1996; 51: 289–296. 10.1093/gerona/51a.6.m289 8914501

[10] Sun R, Guerra R, Shea JB. The posterior shift anticipatory postural adjustment in choice reaction step initiation. Gait Posture. 2015; 41: 894–898. doi: 10.1016/j.gaitpost.2015.03.010 25863873 10.1016/j.gaitpost.2015.03.010

[11] Lee PY, Gadareh K, Bronstein AM. Forward-backward postural protective stepping responses in young and elderly adults. Hum Mov Sci. 2014; 34: 137–146. doi: 10.1016/j.humov.2013.12.010 24569019 10.1016/j.humov.2013.12.010

